# Building resilience: A specialty clinic tailored to older adults at risk for violence and abuse

**DOI:** 10.1177/00912174241272591

**Published:** 2024-08-04

**Authors:** Melba A. Hernandez-Tejada, Deborah M. Little, Madeline J. Bruce, Sarly Butte, Jason Burnett, Leila Wood, Ron Acierno

**Affiliations:** 1 Louis A. Faillace, MD Department of Psychiatry and Behavioral Sciences, 12340 University of Texas Health Science Center at Houston, Houston, TX, USA; 2 Joan and Stanford Division of Geriatric and Palliative Medicine, McGovern Medical School, 12340 University of Texas Health Science Center, Houston, TX, USA; 3 Ralph H. Johnson VA Healthcare System, Charleston, SC, USA

**Keywords:** older adults, ageism, elder mistreatment, PTSD, depression, clinical outcomes, treatment engagement

## Abstract

**Objective:**

Both structural (e.g., ageism) and personal (e.g., stigma) barriers hinder older adults' access to and engagement with mental health care. These barriers are particularly problematic for those vulnerable to interpersonal violence and abuse (e.g., due to social isolation). This study presents a quality improvement program aimed at older adults who have experienced significant stressful events, particularly elder mistreatment, within a larger trauma specialty clinic. Leveraging home-based telemedicine, the clinic provides evidence-based psychotherapy tailored to the needs of older adults.

**Methods:**

From 2021 through 2023, the authors retrospectively examined treatment initiation, engagement, completion, and clinical outcomes among 231 older adults age 60+ who reported trauma that met DSM-5 criterion A criteria for post-traumatic stress disorder, depression, or other mental health comorbid conditions related to their traumatic event. The clinic uses an automated measurement-based care approach that facilitates Quality Improvement projects, allowing the tracking of treatment initiation, engagement, completion, and clinical outcomes for all patients.

**Results:**

The results indicated high treatment completion, high engagement with telemedicine-delivered interventions, and, most importantly, significant changes in clinical outcomes.

**Conclusion:**

These findings highlight the importance of expanding telemedicine-based mental health services for older adults, challenging ageist norms, and prioritizing older adults' mental health needs by providing tailored services to this patient population.

## Introduction

Diminishing stigma and normalizing referral to and support for mental health treatment among older adults continues to pose a persistent challenge, particularly for vulnerable and isolated seniors. Lack of mental health care considerations for older adults is perpetuated by common ageist stereotypes held by many health care providers, including ideas that older adults are an inherently homogeneous group characterized by sadness, loneliness, depression, frailty, dependence on others, and profound physical and intellectual decline.^1^ These ageist stereotypes persistently and negatively influence aging-related mental health policies and practices, including whether someone is referred to mental health care.^2^ Moreover, perpetuating such ageist perspectives may increase morbidity and mortality risks given their physical and mental health consequences and their potential translation into neglect and abuse by others at different levels of society.^3,4^ Taken together, ageism is a significant problem with wide ranging impacts on interpersonal relationships, health services, laws, and local, state, and federal policies.^3,5^

As mentioned, ageism increases risks of long-term physical and mental health problems while diminishing likelihood of referral for these problems.^6^ Health care personnel are not immune to ageist practices.^1^ There appear to be frequent instances of paternalistic or infantilizing approaches by providers toward older patients, such as exclusion from recommended screenings (e.g., screening for domestic violence for younger, but not older adults),^7^ adult protective service (APS) agency investigations, or, perhaps most disturbingly, a dearth of treatment guidelines and offerings that, taken together, reflect a “therapeutic nihilism”.^8^ In the realm of mental health, this nihilism manifests as a lack of will to treat depression or anxiety in older adults, with a de facto normalization of sadness in later life. For older victims of trauma and interpersonal violence, symptoms of PTSD may complement depression, but may also be perceived as normal parts of aging, perpetuated by personal biases and failure to follow the same screening procedures in place for younger adults.^9,10^

Comparing services for interpersonal violence, domestic violence, or family violence between younger and older adults reveals a disparity in access to specialized evidence-based (i.e., effectiveness of treatments demonstrated through replicated, randomized controlled trials). Even when such services are available, referrals often fall short,^11^ and older people use community domestic and family violence services less than their younger counterparts. This is often due in large part to access obstacles. For example, Medicare rules and procedures act to restrict many mental health services, including outdated and inflexible reimbursement schedules, high out of pocket costs for prescription medications that include some antidepressants and antipsychotics in many Part D plans, lack of coverage for some services such as peer support services or psychiatric/psychosocial rehabilitation, mandatory (in most cases) copay requirements, lack of reimbursement for some mental health professionals, lack of Medicare mental health providers, and lack of services customized for older adults.^12-14^ These problems are exacerbated for rural elder victims of violence and mistreatment who experience mental health problems and even poorer coordination of services.

In cases of elder abuse and mistreatment, formalized responses and services are in place, and typically involve law enforcement, APS, and the court system, with some limited involvement from housing entities. However, such formalized responses to elder abuse and mistreatment rarely include mental health or counseling services. Indeed, although Hernandez-Tejada et al^15^ found that targeted promotion of mental health services for older adults significantly increases older adult’s engagement in these and other needed health and social services, scant information is available regarding response of older adults to evidence-based therapies for PTSD and comorbid conditions after a traumatic event like elder mistreatment (EM). This may also result in fewer reported cases to services such as APS or law enforcement compared to their younger counterparts for whom more screening and services are in place,^16^ and reflects the “therapeutic nihilism” highlighted by Nemiroff.^8^

Unfortunately, and perhaps as a result of having received ageist treatment, older adults tend to underreport their symptoms,^17^ contributing to the lack of comprehensive understanding and tailored support and perhaps underrepresentation in mental health research and quality improvement projects.^18^ Normalizing mental health services for older adults, coupled with enhanced accessibility and availability of these services, is crucial to fostering meaningful engagement. For instance, Post-traumatic Stress Disorder (PTSD) has been rarely studied in older adults. This may be due, in part, to the fact that research on interpersonal violence in older adults such as elder mistreatment, particularly with respect to its outcomes, has been widely ignored. Among the limited studies that do exist, PTSD in older adults is strongly associated with recent experiences of violence or abuse.^19-21^

Depression in older adults has a relatively more established research base compared to PTSD. Most older adults report high satisfaction with their lives and achievements and only 2.6% report past year depression, reinforcing the fact that depression is *not* a normal part of aging.^22^ Given that 1 in 10 community residing older adults experience EM *each year*, assessing for the presence of significant and or potentially traumatic event experiences, including signs of abuse and neglect, is justified.^23,24^ Such assessment should, of course, be followed by evidence-based psychological interventions, when appropriate. Notably, existing data suggest a positive association between older age and the completion of such evidence-based treatments,^25^ thus warranting further exploration of opportunities to improve care within this demographic.

Considering older adults and telehealth, the modality has been widely accepted across age groups, particularly following the COVID pandemic. For example, our group showed non-inferiority of the method with older depressed veterans receiving evidence-based psychotherapy.^26^ This modality is particularly useful for those older adults in rural or underserved areas where mental health professionals’ presence is reduced. Moreover, satisfaction with the modality is high. Of course, when older adults (or any potential patients) are not familiar with televideo technology, some efforts to teach them how to use the equipment should be made^27,28^; along with addressing concerns patients might have regarding privacy issues.^29^ Importantly, increased accessibility through telehealth does not increase costs, with evidence showing that telemental health engagement does not increase Medicare expenditures, which is one of the concerns of policymakers.^30^

The primary objective of this quality improvement project was to present psychotherapy retention rates and clinical outcomes of a specialty clinic for older adult survivors of interpersonal violence who were experiencing trauma-related symptoms. There is limited information about trauma related mental health services for older adults, particularly those leveraging technology. This clinic was situated within a larger center dedicated to treating trauma-related sequela and fostering resilience. Opened just before the onset of the COVID-19 pandemic, the older adult clinic was designed to leverage technology to increase reach by offering evidence-based cognitive-behavioral psychotherapy through home-based telemedicine. In other words, the clinic sought to provide effective treatments directly to older adults in their homes, emphasizing adaptability and accessibility in the face of unique transportation challenges commonly faced by older adults, especially those with visual and functional impairment.

## Method

### Quality improvement project methods

To maximize the utility of our QI data and in keeping with best practice clinical standards, we implemented a retrospective chart review following a measurement-based care model (i.e., repeated clinical measures of symptoms related a patient’s mental health condition(s)). Our clinic conducts major assessments before the start of a treatment and at the end of treatment, with monitoring of symptoms weekly, with follow-ups for those who are available to answer our surveys/questionnaires. Data corresponded to patients seeking treatment from 2021 through 2023 who were age 60 and above and reported a trauma that met DSM-5 criterion A criteria for Post-traumatic Stress Disorder (PTSD). We measured treatment initiation and completion rates and collected standardized measures from baseline to completion of treatment of PTSD via the PTSD Module of the MINI-Neuropsychiatric Interview^31^ and the PCL-5^32^ which is a 20-item self-administered questionnaire for monitoring symptoms of PTSD, depression with the PHQ-9,^33^ a highly reliable and valid measure for depression symptom severity, sleep quality using the PSQI^34^ a self-rated questionnaire that assesses sleep quality and disturbances, and anxiety with the GAD-7^35^ which is a valid tool for screening generalized anxiety disorder and assessing its severity. Data were stored in a HIPAA compliant REDCap environment and maintained by the center through the Academic Institution of the University of Texas Health Science Center at Houston.

#### Clinic description and observations

Prior to implementing this specialty clinic for older adult survivors of trauma and interpersonal violence, the larger trauma and resilience counseling center within which the clinic is housed saw approximately 2-3 adults over age 60 for intake assessment (and if appropriate, treatment) per year. The present older adult-focused project was presented to the community via talks and meetings with specific stakeholders in our community, such as senior and community centers, personnel from the office of the Mayor, and adult protective services leadership and social workers. We described our center as a specialty clinic for those over 60 who have experienced a past or recent trauma event that currently impacted their mental health. Clinic mental health professionals were trained in evidence-based therapies for PTSD, depression and other psychological disorders related to trauma exposure. Specific treatments offered in the clinic included Prolonged Exposure Therapy (PE) one of the most efficacious treatments for PTSD, which theoretically based on cognitive behavior principles and empirically, validated with more than 20 years of research background,^36^ Cognitive Processing Therapy (CPT) which is a cognitive behavioral therapy effective in reducing symptoms of PTSD in those who suffer child abuse, combat, rate and natural disasters,^37^ and Written Exposure Therapy (WET) for PTSD a manualized evidence-based therapy that is supported by a fear extinction/emotional processing treatment model,^38^ and Behavioral Activation for depression, one of the most effective treatments for depression based on cognitive behavioral therapy.^39^ Most of these therapies require an average duration of 12-15 weeks. Because the larger trauma and resilience center within which the older adult clinic was housed saw very few older adults in the prior years, specific efforts to promote these new services in the community were made. Meetings with community partners were held through talks, visits to community and senior centers, and pro bono trainings to other health care sites. At these visits, we outlined how our older adult clinic was specifically created for those age 60 and over who had experienced or were experiencing traumatic stress. We stressed the advantages of social support throughout the course of the therapy to improve engagement. Additionally, we trained our therapists and staff to avoid ageist language or phrases (for instance, “you are too old to be acting/doing/having …”); to be aware of methods to deal with cognitive impairment that some older patients may present with, to leverage additional referrals to other services (e.g., meals on wheels) when needed, and to make sure to offer adequate technical support for using telemedicine technology.

## Results

### Clinic population

This clinic excluded patients with significant psychosis, dementia, or substance abuse. Considering specific data on the nature of patients served: the impact of our efforts appeared positive in terms of recruitment. In the first year, over 100 intakes were completed (compared to 2 the prior years), and by October 2023, the clinic had conducted intakes on 231 older adult patients, 74% of whom initiated treatment and 66.3% completed some post treatment data. The average age of these patients was 67.9 years (SD = 6.24 years), most were white (64.1%) or black (26.8%), with significant number of and Hispanic/Latino (13.7%) participants. A majority were female (79.1%). Compared to our patients under 60, those over 60 reported greater numbers of experienced traumatic events (X = 7.19, SD = 4.4 vs X = 7.94, SD = 4.6) (F(11 874) = 5.714, *P <* .05).

Regarding past and recent trauma experience and type of perpetrator, the majority of older adults reported experiencing family members (63.2%) during their childhood. During their young adulthood (age range from 19-39), 23.6% of the older adult patients reported abuse by trusted persons (i.e., family member or friend). Similarly, during their middle-aged adulthood (age range 40-59): 76.36% reported an instance of some type of abuse, but interestingly the majority did not report who the perpetrator was, with only 2%–3% of the total reporting they were victimized by a trusted person. The same refusal to report perpetrator identity was observed for more recent victimization of older adults as well, with only 2%–3% of the total reporting they were abused by a trusted person.

### Treatment completion

Older adults demonstrated excellent treatment completion rates for evidence-based psychotherapy (above 75% compared to 60% of younger patients) and clinical improvements across symptom areas (see below). For comparison, evidence-based psychotherapy trauma treatment completion rate for similar specialty clinics (e.g., VA PTSD clinics) is about 50%.^40,41^ We report the clinical impact of our program in Tables 1 and 2. Specifically, general anxiety as measured by the GAD-7 showed a mean improvement of 3.94 points (t = 8.054, *P* < .001), PTSD as measured by the PCL-5 showed an average improvement of 18.66 points (t = 12.34, *P* < .001); depression as measured by the PHQ-9 showed a mean improvement of 5.13 points (t = 9.48, *P* < .001); and sleep, as measured by the PSQI did not show a mean improvement from baseline to end of treatment. However, there was significant improvement in the PSQI day dysfunction due to sleepiness with average improvement of 1.12 points (t = 11.61, *P* < .001).

**Table 1.** Descriptive statistics for clinical measures: Sleep Quality (PSQI), PTSD (PCL5), Depression (PHQ-9) at baseline and end of treatment (endpoint).

Variable	Mean (N = 171)	SD^a^
GAD-7 baseline	11.73	5.66
GAD-7 endpoint	7.80	5.31
PSQI baseline	10.17	3.74
PSQI endpoint	9.43	4.37
PSQI day dysfunction baseline	1.56	.80
PSQI day dysfunction endpoint	.44	.75
PCL-5 baseline	42.72	16.11
PCL-5 endpoint	24.07	18.23
PHQ-9 baseline	14.17	6.59
PHQ-9 endpoint	9.05	6.55

Abbreviations: GAD: generalized Anxiety Disorder; PSQI: Pittsburgh Sleep Quality Index; PCL: Posttraumatic Stress Disorder Checklist; PHQ: Patient Health Inventory.

^a^Standard Deviation.

**Table 2.** Within-group differences in outcomes from baseline to follow-up.

Paired Samples Test										
		Paired differences					t	df	Significance	
		Mean	SD^a^	S. Error Mean^a^	95% Confidence Interval of the Difference				One-Sided p	Two-Sided p
					Lower	Upper				
	GAD-7 baseline –	3.94	4.46	.49	2.97	4.91	8.05	82	<.001	<.001
	GAD-7 endpoint									
	PSQI baseline –	.733	3.21	.59	−.46	1.93	1.25	29	.110	.220
	PSQI endpoint									
	PSQI day dysfunction baseline – PSQI day dysfunction endpoint	1.12	1.04	.09	.93	1.31	11.61	116	<.001	<.001
	PCL-5 baseline – PCL-5 endpoint	18.66	18.57	1.51	15.67	21.64	12.34	150	<.001	<.001
	PHQ-9 baseline – PHQ-9 endpoint	5.13	6.62	.54	4.06	6.20	9.48	149	<.001	<.001

^a^Standard Deviation; Standard Error Mean.

## Discussion

Findings from this QI project are relevant for older adults who have experienced trauma in the form of interpersonal violence and elder mistreatment, forced social isolation or past domestic violence. First**,** the importance of making mental health services available via home telemedicine to overcome transportation difficulties so commonly affecting older adults cannot be overstated. Similarly, outreach efforts and community partnerships are essential to uptake. Indeed, without these efforts, patients likely would never have been referred to our services. Moreover, evidence-based treatments for trauma sequalae are well tolerated by older adults through this medium, as evidenced by their completion and symptom improvement rates. Currently, such telemedicine delivered, evidence-based therapies to deal with trauma-based symptoms are rarely available for any age group, and this deficit is particularly acute for older adult survivors of trauma and elder mistreatment, for whom post trauma sequalae are rarely assessed. While prevailing stereotypes suggest older adults do not want to engage in telehealth treatment, those over the age of 60 are technology users. In fact, technology is helping older adults to age in place process by addressing issues of connectivity with family, friends, and their health care professionals, including mental health professionals. Our project demonstrated that specialty telehealth clinical services can be successfully and relatively easily implemented with older adults.

Leveraging widely available technology (e.g., tablets, computers, and HIPAA compliant telemedicine software) opens avenues to tailor effective interventions for elder abuse. Indeed, older adults seem responsive to these efforts, as reflected by our increased census, and relatively higher treatment engagement and completion rates for this age group compared to younger age groups. Of note, the success of this specialty trauma clinic for older adults experiencing violence was enhanced by a *pre-pandemic* decision to use telemedicine to increase reach and facilitate access to best practices mental health care. Initial informal feedback indicated that the telemedicine technology was well received, was used by older adults, and implemented before and during the pandemic with no issues, even for cases of severe trauma reactions such as PTSD and depression in older adults. These results align with prior work related to implementation of telemedicine to deliver care for older adults (veterans).^26^

### Limitations and future directions

As this is a QI project in a clinical setting with no experimental controls, observed results may not be generalizable to other settings or populations, including those with similar characteristics. Moreover, many participants failed to regularly complete symptom measures at each opportunity. Additionally, all repeated assessments were self-reports.

Research beyond quality improvement efforts is needed on pragmatic (e.g., primary care-based) assessment and treatments for older adults who have experienced traumatic events and interpersonal violence such as elder abuse. Rigorous experimental protocols are strongly recommended for evaluating effectiveness and impact of these interventions in the older adult population. Specifically, evaluations of evidence based-therapies effective for younger adults should be tested with older populations. Determining which treatments work best is another focus for future research. Once effective assessment and treatments are in hand, identifying the best implementation techniques for these interventions for older adults at risk of abuse and its effects is needed, including information related to broadening access to telemedicine-based services.

Additionally, more research is needed to address specific issues of trust and disclosure. For instance, we observed that older adults were exposed to significant trauma over their lifetime, with a majority reporting an instance of elder abuse to us but not to other authorities. This may be due to stigma related to reporting their loved ones or trusted person as the perpetrators and losing their autonomy.

Finally, implementation studies that also include cost effectiveness assessments of this mode of evidence-based treatment delivery and tailored services for older populations, may impact the future of policymaking in this area. Practical implications of implementing the model presented in this study may elevate the discussion of enhancing accessibility by overcoming logistic barriers, particularly affecting those in areas that are very remote areas, such as those with no access to technology, and/or mental health professionals in their area. Focus also needs to be broadened to improving retention rates, adaptability during crises such as the COVID-19 pandemic, guaranteeing continuity of care, personalization of care, facilitating symptom monitoring and outcomes assessment, and even supporting concerned family members of the older adults by increasing the options the older adults may have to engage in mental health services.

## Data Availability

Deidentified data that support the findings of this study are available upon request from the corresponding author*.
